# Effects of body language training on mental health and performance in adolescent Ju Jutsu athletes

**DOI:** 10.3389/fspor.2026.1868022

**Published:** 2026-06-30

**Authors:** Marc Niering, Jakob Bachmann, Rainer Beurskens, Finn Hansen, Jeremia Rudzki, Johanna Seifert

**Affiliations:** 1 Department of Psychiatry, Social Psychiatry, and Psychotherapy, Hannover Medical School, Hannover, Germany; 2 Institute of Biomechanics and Neurosciences, Nordic Science, Hannover, Germany; 3 Department of Competitive Sports, Brandenburg Ju-Jutsu Federation, Potsdam, Germany; 4 Department of Health and Social Affairs, FHM Bielefeld – University of Applied Sciences, Bielefeld, Germany; 5 Institute of Sport Science, Carl von Ossietzky University of Oldenburg, Oldenburg, Germany; 6 Department of Exercise & Health, Paderborn University, Paderborn, Germany

**Keywords:** competitive anxiety, embodied cognition, emotional regulation, nonverbal behavior, psychological distress

## Abstract

**Background:**

Competitive anxiety and psychological distress are prevalent in youth sports and can impair both mental health and performance. While psychological skills training is established, few interventions target athletes’ body language to reduce anxiety and improve confidence and performance.

**Aims:**

This study examined the effects of a 12-week body language training program on competitive anxiety, psychological well-being, nonverbal behavior, and athletic performance in adolescent Ju-Jutsu athletes.

**Methods:**

Fourteen athletes (14–18 years) were randomly assigned to an intervention (INT) or control group. INT included posture-focused nonverbal behavior training with daily posture exercises, sparring-based practice, and coach feedback. Pre- and post-intervention assessments included the Wettkampfangst-Inventar-State, the Depression Anxiety Stress Scale, video-based body language ratings, and competition performance. Data were analysed using ANCOVAs and mixed ANOVAs.

**Results:**

For performance anxiety, significant group effects at post-test emerged for cognitive anxiety (*p* = .005, *η*^2^ = .53) and somatic anxiety (*p* = .013, *η*^2^ = .44), accompanied by a significant time × group interaction for self-confidence (*p* = .01, *η*^2^ = .43). Regarding psychological distress, significant group effects favored INT for total DASS-21 scores (*p* = .004, *η*^2^ = .23), depression (*p* < .001, *η*^2^ = .33), and stress (*p* = .002, *η*^2^ = .47), while general anxiety showed no significant group effect (*p* = .41, *η*^2^ = .03). Confident body language increased numerically but without significant group differences (*p* = .227, *η*^2^ = .08). Anxious body language decreased over time in both groups, without a significant intervention-specific effect (*p* = .089, *η*^2^ = .06). In competitive performance, INT showed a significant reduction in losses (*p* = .019, *η*^2^ = .41). Improvements in wins (*p* = .06, *η*^2^ = .27) and points per match (*p* = .587, *η*^2^ = .05) were observed within INT but were not supported by significant group-level effects after controlling for baseline values.

**Conclusions:**

In this pilot randomized controlled trial, body language training was associated with reductions in performance anxiety, depressive symptoms, and stress, as well as improvements in selected competitive outcomes. Given the small sample size, these findings should be considered preliminary and require confirmation in adequately powered studies.

## 1 Introduction

The relevance of sport psychology in competitive sports has grown substantially in recent years, with interventions such as self-talk, visualization, and goal setting demonstrating consistent positive effects on performance and psychological outcomes (1). These approaches are widely applied to optimize performance, regulate anxiety and stress, and enhance motivation and self-efficacy (1–3). Their effectiveness in reducing competitive anxiety has been consistently demonstrated (4).

Research further indicates that competitive anxiety is influenced by an interaction of individual psychological characteristics and situational demands, particularly in youth athletes (5). Together with low self-confidence, it represents a central psychological factor that can impair performance (6, 7). Competitive anxiety has also been associated with depressive symptoms (8) and identified as a risk factor for athlete burnout (9). Declining performance, high performance pressure, female sex, participation in individual sports, and injury have been linked to an increased risk of depression (10–13) and anxiety disorders (14, 15). Chronic stress, often triggered by competition-related anxiety, is considered a contributing factor in the development of depressive symptoms in athletes (16).

Such psychological strain can reduce motivation and enjoyment in sports, increasing the risk of dropout (17, 18). Although physical activity has well-documented antidepressant effects (12, 19), depression rates in elite athletes are similar to those in the general population (8, 11, 16, 20, 21). In youth athletes, the prevalence of depressive symptoms may even exceed that of non-athletes (8, 20), although findings remain inconclusive (22). Gwyther et al. (5) reported clinically relevant depressive symptoms in 14% and anxiety symptoms in 8% of elite adolescent male athletes.

This increased vulnerability is likely due to their developmental stage, during which unresolved emotional stress may be harder to regulate due to immature personality structures (23, 24). Young athletes may therefore particularly benefit from psychological support and structured mental training interventions.

Emotional states are often reflected in body language. This includes posture, gesture, facial expressions, and eye contact, all of which play a key role in communication and self-perception (25). Nonverbal behavior influences athletic performance by shaping opponents’ perceptions, affecting their and one's own confidence and composure (26). According to Furley and Roth (27), open, upright, and engaged body language is linked to greater self-confidence and lower anxiety, while slumped and withdrawn postures indicate fear, insecurity, and reduced perceived confidence.

Clinical research further shows that poor posture correlates with anxiety and depressive disorders (28). Elkjær et al. (29) highlight that manipulating posture can enhance the effectiveness of psychotherapy for anxiety and depression, emphasizing the importance of avoiding slouched posture and ensuring congruence between body language and mental state.

According to the facial feedback hypothesis, emotional expressions not only reflect but also modulate feelings by influencing the emotional system (30). Similarly, research on power posing suggests that expansive postures can enhance feelings of power and confidence (31).

Despite growing evidence, the role of body language in sports remains understudied. To our knowledge, research on long-term, sport-integrated body language training programs conducted under real competitive conditions remains limited. Furley and Schweizer (32) note that little is known about how sport-specific nonverbal behavior manifests across disciplines or how it can be systematically trained to appear natural and be retrievable under pressure. They emphasize the need for long-term training that includes education on the function of body language and regular external feedback to enhance athletes’ awareness and behavioral refinement. Based on these gaps, our study investigates whether a 12-week posture-focused body language training in adolescent Ju-Jutsu athletes (a) improves pre-competition body language, (b) reduces competitive anxiety, (c) enhances self-confidence, (d) promotes mental well-being, and (e) improves competitive performance. It is hypothesized that the intervention will strengthen athletes’ nonverbal expression and confidence while reducing performance-related anxiety. In addition, improvements in mental well-being are expected, particularly reductions in subclinical symptoms of depression and stress. However, no significant effects are anticipated in athletes with clinically relevant symptom levels, as posture modification alone cannot replace professional treatment (29).

To test these hypotheses, an experimental design was implemented in which adolescent athletes underwent a structured 12-week intervention program. Its effectiveness was evaluated using self-reported questionnaires, and competitive performance indicators. Methodological details are provided in the following section.

## 2 Material and methods

### 2.1 Experimental design and data collection

The study followed a randomized controlled pre–post design. Baseline assessments were conducted during a regional Ju-Jutsu tournament and comprised both psychological and performance-related measures. Approximately 15–20 min before the first match, athletes completed a questionnaire on competitive anxiety, while their nonverbal behavior in the 30 s preceding the match was video-recorded. To ensure comprehensive assessment, the camera was positioned laterally at the edge of the competition mat, capturing both full-body posture and facial expressions. Competitive performance was evaluated, and mental health symptoms were assessed. The intervention started six days after the baseline assessment and lasted 12 weeks. Post-test assessments were conducted immediately following completion of the intervention period, resulting in an interval of approximately 12 weeks and 6 days between pre- and post-testing.

### 2.2 Participants

To determine the required sample size, an *a priori* power analysis was conducted using G*Power 3.1 (33). As no prior studies have specifically examined the effects of body language training in sports, the study by Marshall and Gibson (34) was used as a reference. This study investigated the effects of a 4-week imagery training on competitive anxiety, self-confidence, and athletic performance in adolescent acrobatic gymnasts, reporting effect sizes ranging from *η*^2^ = .10 to *η*^2^ = .46. For the current calculation, the lower bound (*η*^2^ = .10) was chosen as a conservative estimate, corresponding to a medium effect size of *f* = 0.33 (35). Based on *α* = .05, power = .95, two groups, and two measurement points, the required sample size was *n* = 32. Because only 14 athletes were retained, the study is substantially underpowered and all statistical inferences should be interpreted with caution due to increased Type II error risk and limited generalizability. Accordingly, our study should be considered a pilot randomized controlled trial with an exploratory focus rather than a confirmatory design.

Initially, 20 athletes were recruited (17 male, 3 female). Due to illness and injury, six athletes dropped out before baseline assessment. The final sample consisted of 14 athletes who were assigned to an intervention group (INT) or a control group (CON) using a stratified matched-pair randomization procedure. Participants were first stratified by sex and then paired based on similar self-rated athletic performance levels. Within each pair, one athlete was randomly allocated to the intervention group and the other to the control group using the online platform randomizer.org (36). Randomization was performed by a member of the research team (J.S.) who was not involved in outcome assessment. Allocation concealment was not implemented due to the small sample size and the logistical requirements of the intervention.

As shown in Table 1, both groups were comparable in age, training experience, training load, and self-rated athletic success. All participants were members of the regional Ju-Jutsu squad and regularly competed at the national level. Based on their competition activity, training volume, and experience, they met criteria for high-performance sport as defined by Swann et al. (37).

**Table 1:** Participant characteristics.

Variable	Total	INT	CON
N (sex)	14 (2 F, 12 M)	7 (6 M, 1 F)	7 (6 M, 1 F)
Age (years)	15.29 ± 1.20	15.29 ± 1.11	15.29 ± 1.38
Training experience (years)	8.43 ± 1.34	8.71 ± 1.80	8.14 ± 0.69
Training load (h/week)	7.93 ± 1.27	8.29 ± 1.50	7.57 ± 0.98
Self-rated athletic success	3.71 ± 0.73	4.14 ± 0.69	3.29 ± 0.49

Self-rated athletic success was assessed on a 5-point Likert scale (1 = “not successful at all”, 5 = “very successful”). Values represent means ± standard deviations.

Participant recruitment was conducted in collaboration with the Brandenburg and Lower-Saxony Ju-Jutsu Federations, which provided a list of eligible athletes. Participation was voluntary, and written informed consent was obtained from all athletes and/or their legal guardians prior to the start of the study.

Inclusion criteria comprised active involvement in competitive-level Ju-Jutsu, membership in the regional squad, national competition experience, and an age between 14 and 18 years. Exclusion criteria included acute injuries or chronic conditions that could interfere with training participation, ongoing psychotherapeutic treatment, and the absence of valid informed consent. None of the participants had previous experience with structured body language or posture-focused training.

### 2.3 Psychological and performance measures

#### 2.3.1 Assessment of state performance anxiety

To assess state-related psychological variables, two validated self-report instruments were used. Competitive state anxiety was measured using the German Wettkampfangst-Inventar-State (WAI-S) (38). The WAI-S consists of 12 items, each rated on a 4-point Likert scale ranging from 1 (“not at all”) to 4 (“very much”), and covers three subscales: somatic anxiety, cognitive anxiety (worry), and self-confidence, with four items per dimension.

This instrument enables a differentiated assessment of pre-competition anxiety. The WAI-S has been applied in previous studies (39, 40) and has demonstrated satisfactory internal consistency, with Cronbach's alpha values ranging from .79 to .82 (38).

#### 2.3.2 Assessment of symptoms of depression, anxiety and stress

Symptoms of depression, anxiety, and stress were assessed using the Depression Anxiety Stress Scales—Short Form (DASS-21) (41).

The DASS-21 allows for the evaluation of changes in psychological distress over time and the identification of clinically relevant symptom levels in adolescents. According to Nilges and Essau (41), subscale thresholds for clinical concern are 10 for depression, 6 for anxiety and 10 for stress, to achieve balanced sensitivity and specificity.

Internal consistency of the subscales is high, with Cronbach's alpha ranging from 0.91 for depression, 0.78–0.82 for anxiety, and 0.81–0.89 for stress (41), supporting the reliability of the instrument.

In addition to the three subscales, the total DASS-21 score was also used in our study to provide a broader measure of general psychological distress. Recent research suggests that, particularly in adolescents, the distinct subscales may not always show sufficient psychometric differentiation, whereas the total score demonstrates greater overall stability (42, 43).

#### 2.3.3 Assessment of body language

In addition to psychological measures, athletes’ body language was recorded during the final 30 s before each match using video footage. Two independent raters evaluated the athletes’ nonverbal behavior along two dimensions: “confident” and “anxious” body language.

Confident body language was defined as dominant, open, and controlled posture, as taught through the target postures in the intervention. Raters focused on posture, gaze direction, facial expression, and behavioral calmness and control. In contrast, anxious body language was characterized by closed, slouched posture and nervous, avoidant behavior. Inter-rater reliability was excellent for confident body language (ICC = .89) and good for anxious body language (ICC = .81).

Evaluations were conducted using a 7-point Likert scale. A score of 1 represented very low confidence or low anxiety expression, while a score of 7 indicated a highly confident or highly anxious demeanor. The rating procedure was adapted from the methodology of Cuddy et al. (44), in which independent raters assessed nonverbal presence, including perceived confidence, using a similar Likert-based scale. A global rating approach was selected because the aim was to assess overall impressions of body language rather than discrete behavioral components as coded by Furley and Roth (27). Due to the nature of the intervention, no blinding of participants or coaches was possible. Raters were not blinded to group allocation. Prior to the rating procedure, both raters completed a standardized familiarization and calibration process using example videos.

#### 2.3.4 Assessment of competitive performance

Competitive performance was assessed based on the number of wins and losses, as well as the athletes’ average score per match, following established approaches in performance evaluation (45, 46). Including both indicators reduces the limitations of relying solely on match outcomes, which may be influenced by uncontrollable external factors.

### 2.4 Experimental intervention

The intervention was designed as a posture-focused nonverbal behavior training program. The intervention began with a 60-min theoretical introduction. This session covered the role of body language in competitive contexts, its connection to internal emotional states, and its influence on opponents (27). In line with embodied cognition frameworks, the intervention was grounded in the assumption that bodily states can shape emotional regulation, perceived control and competitive presence. Expansive, upright and assertive postures are associated with increased confidence and reduced anxiety, whereas contractive postures tend to reinforce threat perception and self-focused attention (29, 47). This theoretical perspective informed the structure and execution of all posture components practiced in the intervention. The components of the target posture were explained and rehearsed in practice. Central elements included a dominant, open, and upright stance with shoulders slightly retracted and chest lifted (27, 29). Facial expressions were to appear calm and confident (48), and eye contact was directed toward the opponent or referee (27).

The arms were positioned openly, either relaxed at the sides or placed on the hips, with movements kept controlled and steady to avoid crossed postures or nervous gestures (27).

This session marked the beginning of a 12-week intervention period. During this phase, INT athletes practiced the target body posture daily for 60 s in front of a mirror. Additionally, they applied the posture twice weekly before ten sparring matches per session. Coaches provided regular feedback on posture execution (27). Training adherence was documented in a self-report protocol in which participants recorded daily completion of the exercises. Mean adherence to the intervention was 82.4% ± 10.7%, based on participants’ self-report logs.

CON continued their regular Ju-Jutsu training routines, including their usual technical, tactical, and physical training sessions. They did not receive any body language-related instructions, additional educational sessions, or placebo intervention.

Post-intervention assessments mirrored the baseline procedure and were again conducted during a regional tournament. Participants completed the WAI-S 15–20 min before their first match, their body language was video-recorded, and match data were collected. The DASS-21 was completed the following morning under the same conditions as at baseline because the instrument assesses symptoms of psychological distress over the previous week rather than acute competition-related states. This timing was chosen to minimize the potential influence of immediate post-competition emotions and match outcomes on participants’ responses.

### 2.5 Statistical analysis

Statistical analyses were performed using SPSS Statistics (Version 29.0.2.0, IBM Corp., Armonk, NY, USA). Assumptions for parametric testing were examined using the Shapiro–Wilk test for normality and Levene's test for homogeneity of variances. In addition, the assumption of homogeneity of regression slopes was tested to determine the appropriateness of analysis of covariance (ANCOVA).

For outcomes in which this assumption was met, post-test scores were compared between groups using ANCOVA with baseline values entered as covariates (49). For outcomes in which the assumption was violated, 2 × 2 mixed ANOVAs were conducted with one within-subject factor (time: pre vs. post) and one between-subject factor (group: intervention vs. control). The homogeneity of regression slopes assumption was met for somatic anxiety, cognitive anxiety, depression, anxiety, confident body language, losses, and points per match. Self-confidence and wins violated this assumption and were therefore analyzed using mixed ANOVAs.

To illustrate within-group changes over time, additional paired *t*-tests were conducted for normally distributed samples and Wilcoxon signed-rank tests for non-normally distributed samples. When the Wilcoxon test was not applicable, sign tests were used. For between-group comparisons, effect sizes were reported as partial eta squared (*η*^2^). Interpretation of effect sizes followed conventional thresholds (35): small (*η*^2^ ≥ .01, *d* ≥ .20, *r* > .30), medium (*η*^2^ ≥ .06, *d* ≥ .50, .30 ≤ *r* < .50), and large effects (*η*^2^ ≥ .14, *d* ≥ .80, *r* > .50). Given the exploratory nature of this pilot trial, no adjustment for multiple comparisons was applied. The analyses were intended to identify potentially relevant effects to be examined in future adequately powered confirmatory studies.

## 3 Results

### 3.1 Performance anxiety

**Figure 1:**
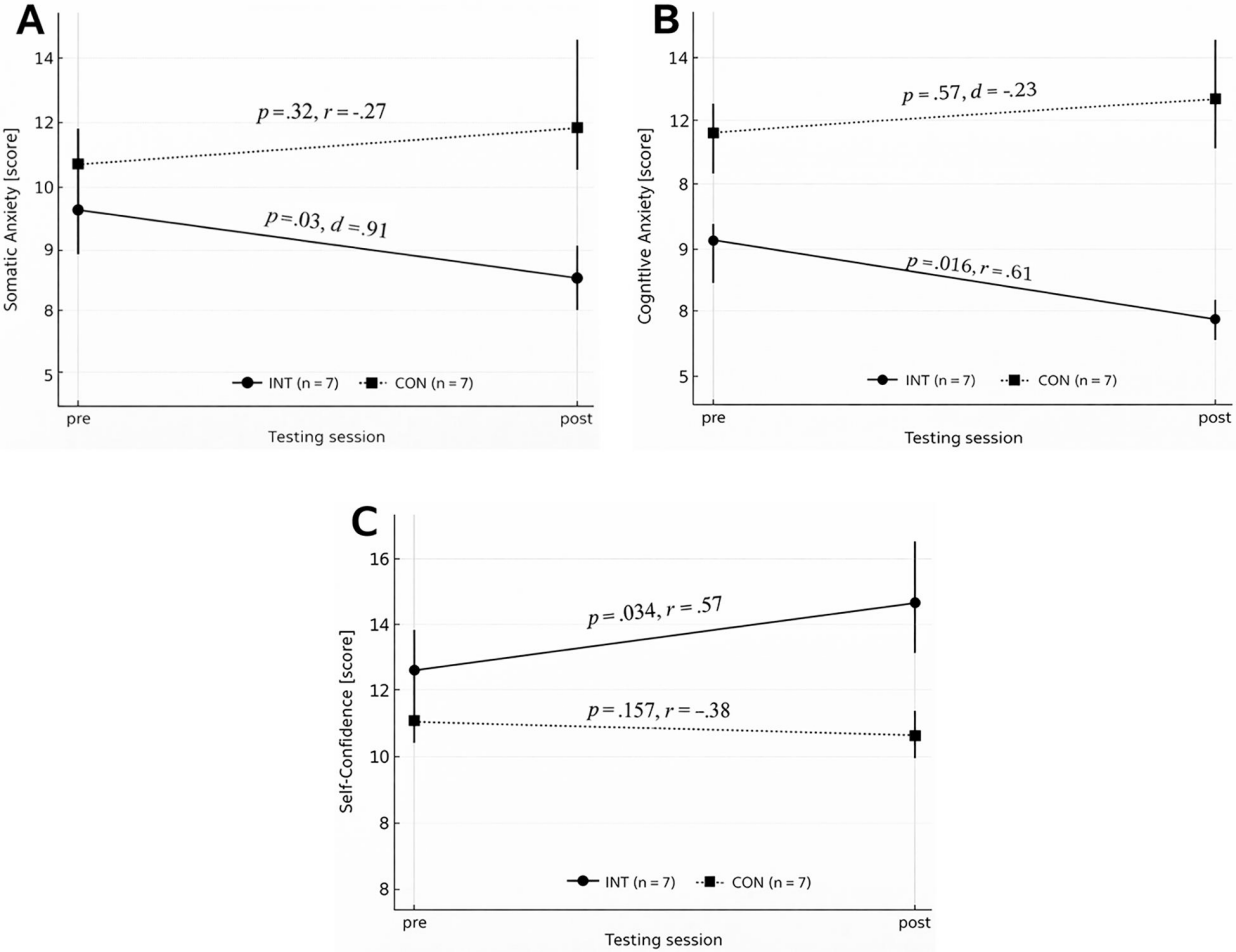
WAI-S somatic anxiety **(A)**, cognitive anxiety **(B)**, and self-confidence **(C)** scores across pre- and post-intervention. Means and standard errors of the mean (SEM) are shown.

Figures 1A–C illustrate the trajectories of self-confidence, cognitive anxiety, and somatic anxiety across pre- and post-test.

For self-confidence, the 2 × 2 mixed ANOVA revealed a significant time × group interaction [*F*(1,12) = 16.34, *p* = .01, *η*^2^ = .43]. Paired comparisons indicated a significant pre–post increase in INT (*p* = .034, *r* = .57), whereas CON showed no significant change (*p* = .157, *r* = −.38).

For cognitive anxiety, ANCOVA with baseline values entered as covariates revealed a significant group effect at post-test [*F*(1,11) = 12.36, *p* = .005, *η*^2^ = .53]. Adjusted means indicated lower anxiety in INT compared to CON. In line with this, Figure 1B shows that cognitive anxiety decreased significantly from pre- to post-test in INT (Δ*M* = −2.43, *p* = .016, *r* *=* .61), while no significant change occurred in CON (Δ*M* = .43, *p* = .57, *d* = −.23).

For somatic anxiety, ANCOVA also revealed a significant group effect at post-test [*F*(1,11) = 8.72, *p* = .013, *η*^2^ = .44], with lower adjusted post-test scores in INT compared to CON. Descriptively, INT did show a significant reduction [Δ*M* = −1.71, 95% CI(−.03, 3.46), *p* = .03, *d* = .91], while CON remained stable (*p* = .32, *r* = −.27).

### 3.2 Symptoms of depression, anxiety and stress

**Figure 2:**
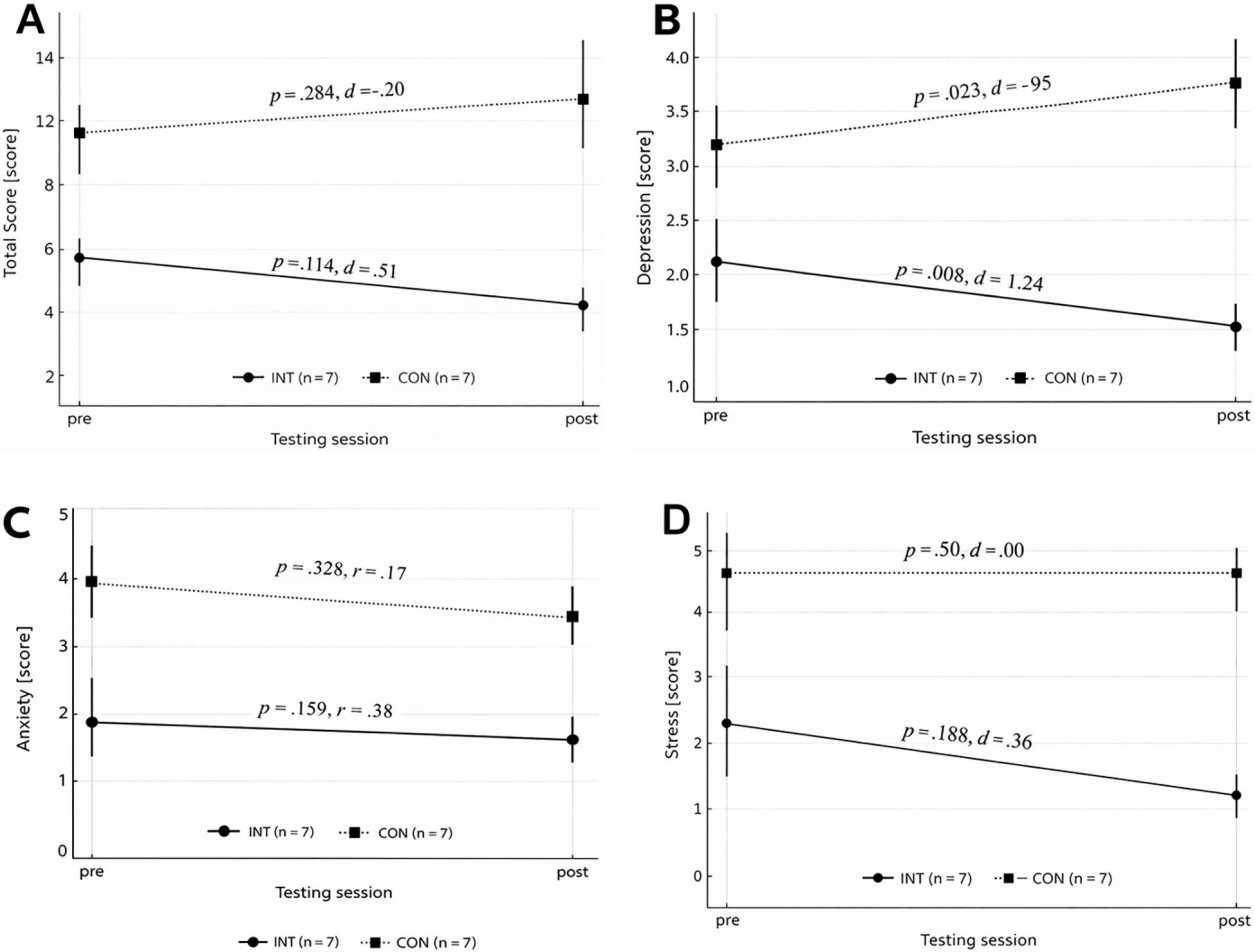
DASS-21 total score **(A)**, depression **(B)**, anxiety **(C)**, and stress **(D)** across pre- and post-intervention. Means and SEM are shown.

Figures 2A–D illustrate the trajectories of DASS-21 total score, depression, anxiety, and stress across pre- and post-test.

For the total score, INT showed a reduction from pre- to post-test (Δ*M* = −2.14), whereas CON remained essentially unchanged (Δ*M* = .29). Within-group changes were not significant (INT: *p* = .114, *d* = .51, 95% CI [−.30, 1.28]; CON: *p* = .284, *d* = −.20, 95% CI [−.97, −.53]. The ANCOVA revealed a significant main effect of group, indicating lower post-intervention total DASS-21 scores in the intervention group compared to the control group after controlling for baseline values [*F*(1,11) = 13.44, *p* *=* .004, *η*^2^ *=* .23].

For depression, scores decreased in INT (Δ*M* = −.86) and increased in CON (Δ*M* =  + .71). The within-group pre–post change decreased significantly in INT (*p* = .008, *d* = 1.24, 95% CI [.21, 2.23) while increasing significantly in CON (*p* = .023, *d* = −.95, 95% CI [−1.83, −.01). ANCOVA with baseline as covariate indicated a clear group effect at post-test [*F*(1,11) = 22.05, *p* < .001, *η*^2^ = .33].

For anxiety, both groups showed small decreases (INT: Δ*M* = −.29, *p* = .159, *r* = −.38; CON: Δ*M* = −.43, *p* = .328, *r* = .17). This pattern was not supported by ANCOVA [*F*(1,11) = 0.75, *p* = .41, *η*^2^ = .03].

For stress, INT showed a numerical decrease [Δ*M* = −1.00, 95% CI (−.42, 1.12), *p* = .188, *d* = .36], whereas CON remained stable [Δ*M* = .00, 95% CI (−.74,.74), *p* = .50, *d* = .00]. The ANCOVA revealed a significant main effect of group on post-intervention scores after controlling for baseline values [*F*(1,11) = 17.47, *p* = .002, *η*^2^ = .47].

### 3.3 Body language

**Figure 3:**
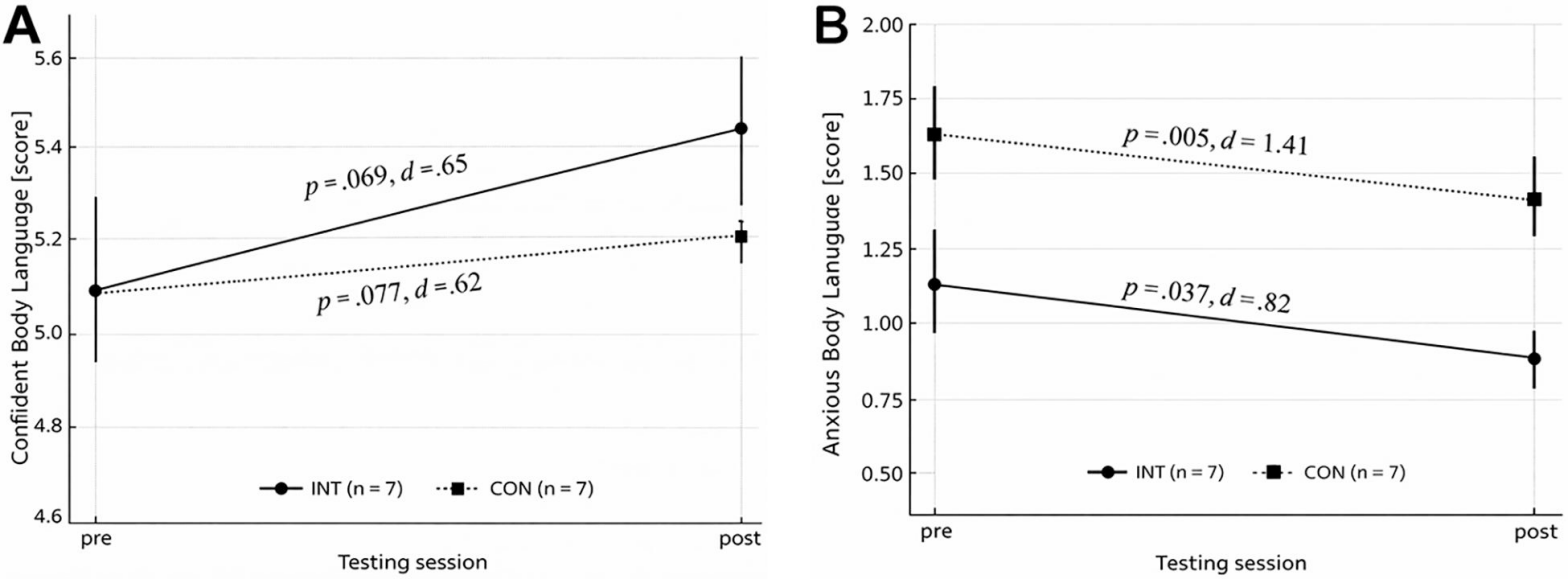
Confident body language **(A)** and anxious body language **(B)** scores across pre- and post-intervention. Means and SEM are shown.

Figures 3A,B illustrate the trajectories of self-confident and anxious body language across pre- and post-test.

For self-confident body language, INT increased from pre- to post-test (INT: Δ*M* =  + .43), and CON showed a smaller increase (CON: Δ*M* = .18). Within-group changes did not reach significance [INT: *p* = .069, *d* = .65, 95% CI (−.19, 1.05); CON: *p* = .077, *d* = .62, 95% CI (−.09, .45)]. ANCOVA with baseline as covariate indicated no significant group effect at post-test [*F*(1,11) = 1.64, *p* = .227, *η*^2^ = .08].

For anxious body language, the intervention group showed a significant decrease from pre- to post-assessment [INT: Δ*M* = −.29, 95% CI (−.62, −.04), *p* = .037, *d* = .82]. A comparable and also significant decrease was observed in the control group (CON: Δ*M* = −.29, *p* = .005, *d* = 1.41). However, the ANCOVA revealed no significant main effect of group after controlling for baseline values [*F*(1,11) = 3.49, *p* = .089, *η*^2^ = .06].

### 3.4 Competitive performance

**Figure 4:**
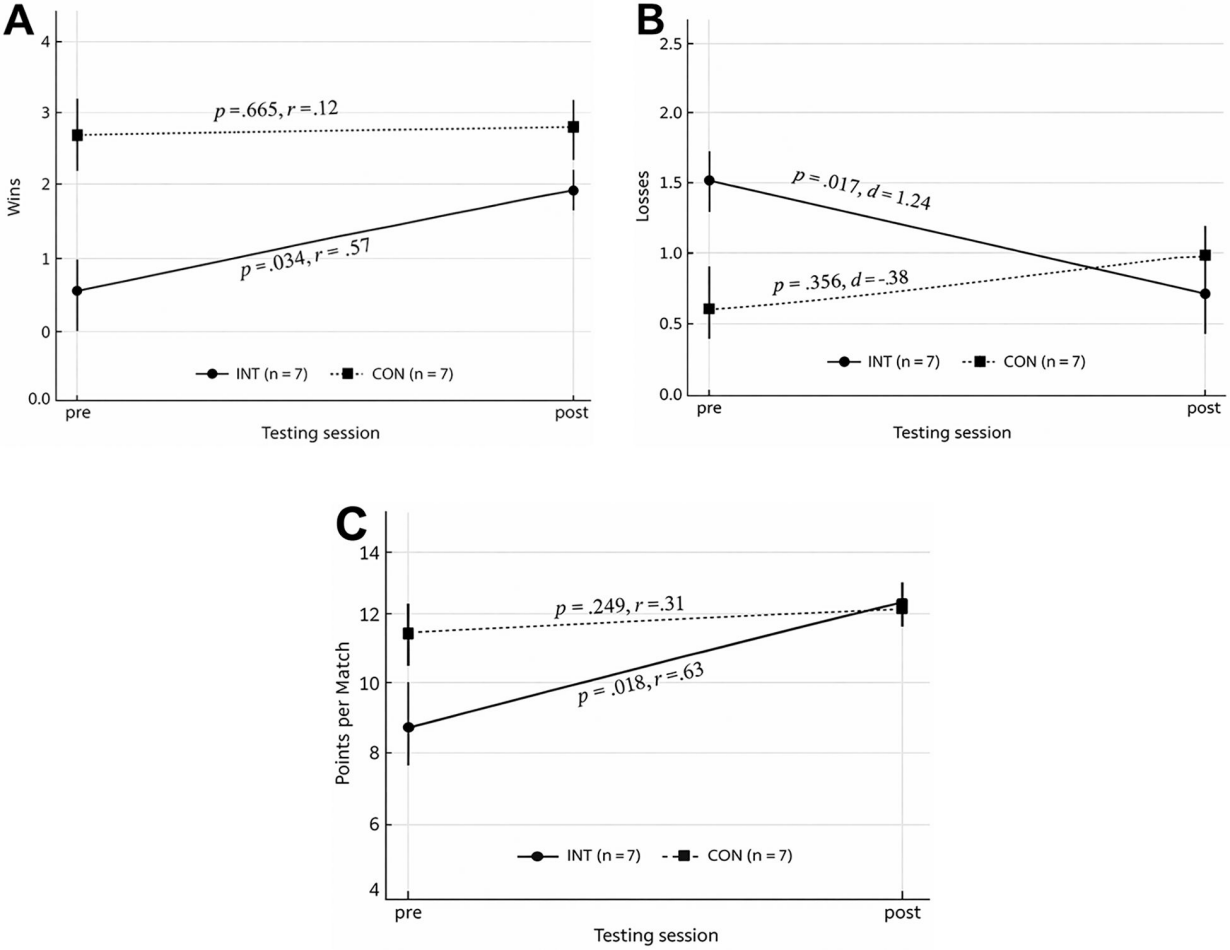
Wins **(A)**, losses **(B)**, and points per match **(C)** across pre- and post-intervention. Means and SEM are shown.

Figures 4A–C show the competitive outcomes in terms of wins, losses, and points per match.

For wins (Figure 4A), the 2 × 2 mixed ANOVA revealed a near significant time × group interaction [*F*(1,12) = 4.324, *p* = .06, *η*^2^ = .265]. However descriptively, the paired comparisons showed a significant increase for INT (Δ*M* = 1.14, *p* = .034, *r* = .57), whereas CON remained stable (Δ*M* = −.14, *p* = .665, *r* = .12).

For losses (Figure 4B), INT decreased from pre- to post-test (Δ*M* = .86, *p* = .017, *d* = 1.24), whereas CON slightly increased (Δ*M* = .29, *p* = .356, *d* = −.38). This group difference was confirmed by the ANCOVA [*F*(1,11) = 7.487, *p* = .019, *η*^2^ = .41].

For points per match (Figure 4C), INT significantly increased from pre- to post-test (Δ*M* = 3.43, *p* = .018, *r* = .63), while CON showed only a slight non-significant improvement (Δ*M* = .77, *p* = .249, *r* = .31). However, when controlling for pre-test scores the ANCOVA did not reveal a significant effect [*F*(1,11) = .460, *p* = .587, *η*^2^ = .051].

## 4 Discussion

The aim of this study was to examine the effects of a structured 12-week body language training program on competitive state anxiety, psychological distress, and performance outcomes in adolescent Ju-Jutsu athletes. Given the exploratory nature of this pilot trial and the limited sample size, the findings should be interpreted with caution and viewed as preliminary evidence rather than definitive confirmation of intervention effects.

INT showed statistically significant differences in cognitive and somatic performance anxiety as well as self-confidence, whereas general anxiety did not change. Depression decreased significantly within INT, and group differences were observed for stress and overall psychological distress. Body language showed changes over time, but these were not clearly intervention-specific. Confident body language increased numerically without reaching statistical significance, and anxious body language decreased in both groups. Competitive performance outcomes were mixed, as INT showed a statistically significant reduction in losses and within-group increases in points per match, while other indicators did not show consistent group-level effects. This discrepancy between psychological improvements and the lack of clear changes in body language suggests that the underlying mechanisms of the intervention require further clarification.

### 4.1 Effects on performance anxiety

The observed increases in self-confidence and reductions in cognitive and somatic anxiety suggest meaningful changes in performance-related psychological states. Taken together, these findings indicate differences in performance anxiety between groups. These findings are consistent with previous research demonstrating that psychological interventions can improve emotional regulation and reduce competitive anxiety in athletes (4).

The concurrent reduction in anxiety and increase in confidence suggest improvements in psychological states (50). Further, the differential effects observed across anxiety dimensions may suggest that somatic and cognitive components of performance anxiety may be influenced through partially distinct mechanisms, which is consistent with embodiment models (47) and the facial feedback hypothesis (51). Prior studies have suggested that adopting expansive postures, often referred to as power posing, may enhance perceived control and reduce stress responses, although recent evidence suggests that such effects are small and inconsistent (31). Moreover, a meta-analysis by Coles et al. (30) suggests that nonverbal expressions such as posture and facial feedback may influence emotional states, although the magnitude of these effects is generally small and variable.

### 4.2 Effects on symptoms of anxiety, depression and stress

The observed reductions in psychological distress, particularly in depression and stress, suggest a potential beneficial effect of the intervention on mental well-being. These findings suggest an association between the intervention and reductions in psychological distress, particularly in depressive and stress-related symptoms, while general anxiety symptoms were less consistently affected. One possible explanation is provided by Elkjær et al. (29), who demonstrated that posture-based interventions can reduce symptoms of depression and anxiety, especially in subclinical populations. The absence of robust effects for anxiety in our study contrasts with findings by Weineck et al. (52), who reported a significant decrease in general anxiety symptoms after a two-week power posing intervention in university students. This discrepancy may be explained by differences in sample characteristics, intervention duration, or the focus of anxiety assessment. These findings are in line with previous research indicating that posture-based interventions can influence affective states, particularly in subclinical populations (29). Whereas the DASS-21 primarily captures general, trait-like anxiety, our intervention was designed to target emotion regulation in performance-relevant contexts, which may partly account for the weaker effects on generalized anxiety symptoms.

Further evidence supports the relationship between posture and affective states. Asadi Melerdi et al. (53) found associations between postural abnormalities and higher distress in adolescents, while Wilkes et al. (54) showed that upright posture can improve affective experience in individuals with depressive symptoms. Taken together, the findings suggest that body language training may function as a low-threshold intervention to reduce depressive and stress-related symptoms, even in non-clinical populations, while its effects on generalized anxiety appear more limited.

### 4.3 Effects on body language

Changes in body language were observed over time, but these were not clearly attributable to the intervention. Overall, the results provide preliminary evidence of changes in body language over time, but do not support a clear intervention-specific effect. This discrepancy suggests that the observed psychological effects may be driven less by measurable changes in posture and more by factors such as increased self-awareness, attentional focus, or perceived control, which is in line with existing theoretical frameworks on embodied cognition and self-regulation (29, 30). This interpretation suggests that the intervention may primarily operate through internal cognitive and attentional processes rather than through observable changes in nonverbal behavior.

Although no intervention-specific effects were observed, the observed changes over time are compatible with theoretical assumptions by Furley and Schweizer (32), who proposed that controlled, confident body language can become accessible under stress if it has been deliberately practiced beforehand. This extends prior psychological research on power posing, which has primarily investigated short-term effects on self-perception and affect in laboratory settings (44).

In contrast, our study employed a twelve-week training protocol integrated into athletes’ regular practice routines and applied directly in real-world competitive settings. As such, changes in body language observed in our study may reflect increased familiarity, habituation, or general adaptation effects, rather than robust intervention-specific learning effects. The findings therefore highlight both the potential and current limitations of embodiment-based approaches in high-performance sports.

At present, no comparable sport-specific intervention studies on body language exist, which limits direct comparison. Nonetheless, this study provides an important initial contribution to the field and offers a valuable starting point for future investigations on the effectiveness of nonverbal training methods in competitive sports.

### 4.4 Effects on competitive performance

The intervention was associated with selective changes in competitive performance, particularly a reduction in losses, while other performance indicators showed less consistent effects. Taken together, these findings indicate that the intervention was associated with changes in selected performance outcomes, particularly reductions in losses, while effects on wins and points per match were less consistent at the group level. This pattern suggests that the intervention may have influenced specific aspects of performance rather than leading to uniform improvements across all indicators. Thus, competitive performance appears to have been influenced in a differentiated manner rather than showing a uniform improvement across all indicators.

One possible explanation lies in the combination of emotional and behavioral changes. The observed reductions in performance anxiety and increases in confidence may have contributed to a more focused and assertive presence during competition. According to Lochbaum et al. (6), these factors are among the key psychological predictors of athletic performance. Body language training may contribute to both internal regulation and externally observable behavior, thereby influencing competitive behavior.

In addition, more confident body language may influence opponents’ perceptions. Previous studies have shown that dominant nonverbal cues such as upright posture, steady eye contact, and calm movement patterns can influence how opponents assess an athlete's assertiveness, concentration, and mental strength (25). Buscombe et al. (55) found that experienced tennis players who viewed short video clips of opponents displaying either dominant or submissive body language perceived those with dominant body language as more assertive and capable. Similarly, Fritsch et al. (56) reported that observers were able to accurately assess the affective states of amateur tennis players based on their nonverbal behavior following competitive points, particularly after lost points, highlighting the impact of visible emotional expressions in sport. Such impressions can reduce an opponent's own confidence and lead to more cautious behavior, which may contribute to competitive outcomes (32).

Additional evidence on the performance relevance of nonverbal signals comes from Kraus and Chen (48), who observed that intense smiling during eye contact one day before a professional MMA fight was associated with lower likelihood of winning. The authors interpreted this behavior as a sign of reduced dominance, which may negatively affect motivation and opponent perception. Although our study did not include a specific analysis of facial expressions, these findings illustrate that even subtle nonverbal signals, whether consciously or unconsciously expressed, may influence competitive behavior and success.

This influence of minimal body signals is also supported by experimental studies showing that brief nonverbal impressions prior to performance are sufficient to draw conclusions about an athlete's capabilities (57). Furthermore, Furley and Schweizer (32) reported that under stress, even minor postural cues can be perceived as signs of uncertainty. Accordingly, training a controlled and confident body language may have contributed to changes in competitive dynamics, particularly by reducing maladaptive behaviors and errors, which may help explain the observed reduction in losses.

### 4.5 Limitations

The interpretation of our findings must take into account several methodological limitations. First, the study should be interpreted as a pilot randomized controlled trial due to the substantially reduced sample size relative to the *a priori* calculation. Most importantly, the sample size was very small (*n* = 14), falling well below the *a priori* calculated requirement (*n* = 32), which substantially limits statistical power and increases the likelihood of type II errors. While large effect sizes were observed across several variables, many failed to reach statistical significance, suggesting limited statistical power to detect effects. In addition, multiple outcomes were analyzed without adjustment for multiple comparisons, which increases the risk of Type I errors.

The field-based assessment in real competitive settings involved various uncontrollable factors such as opponent strength, referee decisions, and venue variability, which may have influenced outcomes. In addition, body language ratings by two independent raters, although based on predefined criteria, remain partly subjective due to the absence of a validated coding system for martial arts. The applied rating approach was developed specifically for this study and conceptually informed by theoretical considerations on power posing (44). However, it should be noted that no standardized or validated coding system was directly adapted, which limits comparability and generalizability. The rating approach was also not validated against established sport-specific coding systems. Furthermore, no blinding was implemented for participants, coaches, or raters, which introduces potential performance and detection bias and may have influenced both subjective and observer-based outcomes. As a result, expectancy effects may have influenced self-reported outcomes such as competitive anxiety and psychological distress, while knowledge of group allocation may have affected observer-based ratings of body language. In addition, the use of a passive control group limits the ability to distinguish intervention-specific effects from non-specific factors such as increased attention, expectancy effects, or placebo-like influences. Moreover, no manipulation check was conducted to verify whether the intervention effectively altered participants’ body language as intended.

### 4.6 Practical implications

The findings suggest that body language training may represent a simple and low-cost addition to existing psychological support strategies in youth sports. Practical applications include low-threshold strategies such as mirror exercises, video feedback, and posture coaching, which can be integrated into regular training routines to enhance emotional regulation and competitive presence. Coaches and sport psychologists may use these approaches to increase athletes’ awareness of their nonverbal behavior and to support the development of self-confidence in competitive situations.

## 5 Conclusions

This study examined the effects of a 12-week body language training program on performance anxiety, psychological distress, body language, and athletic performance in adolescent Ju-Jutsu athletes. The intervention was associated with reductions in cognitive and somatic anxiety, depression, and stress, while general anxiety showed no significant intervention-related effects. Changes in confident and anxious body language were observed over time, but without clear intervention-specific group effects. Athletes in the intervention group demonstrated a significant reduction in losses, whereas other competitive performance indicators showed mixed or non-significant group-level effects. Despite the small sample size, several medium-to-large effect sizes suggest potential practical relevance of the program.

The findings support theoretical frameworks proposing that body language not only reflects but may also influence internal states and social perception in competitive contexts.

Future research should replicate these findings in larger, gender-balanced samples and investigate long-term effects. Comparative studies across different sports and intervention durations may help clarify underlying mechanisms and optimize implementation.

Overall, structured body language training may represent a promising complementary approach for adolescent athletes. However, given the pilot character of the study and the limited sample size, the findings should be considered preliminary until replicated in larger and adequately powered trials.

## Data Availability

The raw data supporting the conclusions of this article will be made available by the authors, without undue reservation.
